# Associations between COVID‐19 and hospitalisation with respiratory and non‐respiratory conditions: a record linkage study

**DOI:** 10.5694/mja2.51778

**Published:** 2022-11-14

**Authors:** Stacey L Rowe, Karin Leder, Kylie Dyson, Lalitha Sundaresan, Dennis Wollersheim, Brigid Lynch, Ifrah Abdullahi, Benjamin C Cowie, Nicola Stephens, Terence M Nolan, Sheena G Sullivan, Brett Sutton, Allen C Cheng

**Affiliations:** ^1^ Monash University Melbourne VIC; ^2^ Victorian Department of Health Melbourne VIC; ^3^ Royal Melbourne Hospital Melbourne VIC; ^4^ Mathematica Oakland CA United States of America; ^5^ Cancer Epidemiology Centre Cancer Council Victoria Melbourne VIC; ^6^ Melbourne School of Population and Global Health Melbourne VIC; ^7^ Latrobe University Melbourne VIC; ^8^ WHO Collaborating Centre for Viral Hepatitis the Peter Doherty Institute for Infection and Immunity Melbourne VIC; ^9^ University of Tasmania Hobart TAS; ^10^ Peter Doherty Institute for Infection and Immunity at the University of Melbourne Melbourne VIC; ^11^ Murdoch Children's Research Institute Melbourne VIC; ^12^ WHO Collaborating Centre for Reference and Research on Influenza the Peter Doherty Institute for Infection and Immunity Melbourne VIC

**Keywords:** COVID‐19, Epidemiology, Public health, Public health, SARS‐COV‐2, Hospitals

## Abstract

**Objectives:**

To assess associations between SARS‐CoV‐2 infection and the incidence of hospitalisation with selected respiratory and non‐respiratory conditions in a largely SARS‐CoV‐2 vaccine‐naïve population .

**Design, setting, participants:**

Self‐control case series; analysis of population‐wide surveillance and administrative data for all laboratory‐confirmed COVID‐19 cases notified to the Victorian Department of Health (onset, 23 January 2020 – 31 May 2021; ie, prior to widespread vaccination rollout) and linked hospital admissions data (admission dates to 30 September 2021).

**Main outcome measures:**

Hospitalisation of people with acute COVID‐19; incidence rate ratios (IRRs) comparing incidence of hospitalisations with defined conditions (including cardiac, cerebrovascular, venous thrombo‐embolic, coagulative, and renal disorders) from three days before to within 89 days of onset of COVID‐19 with incidence during baseline period (60–365 days prior to COVID‐19 onset).

**Results:**

A total of 20 594 COVID‐19 cases were notified; 2992 people (14.5%) were hospitalised with COVID‐19. The incidence of hospitalisation within 89 days of onset of COVID‐19 was higher than during the baseline period for several conditions, including myocarditis and pericarditis (IRR, 14.8; 95% CI, 3.2–68.3), thrombocytopenia (IRR, 7.4; 95% CI, 4.4–12.5), pulmonary embolism (IRR, 6.4; 95% CI, 3.6–11.4), acute myocardial infarction (IRR, 3.9; 95% CI, 2.6–5.8), and cerebral infarction (IRR, 2.3; 95% CI, 1.4–3.9).

**Conclusion:**

SARS‐CoV‐2 infection is associated with higher incidence of hospitalisation with several respiratory and non‐respiratory conditions. Our findings reinforce the value of COVID‐19 mitigation measures such as vaccination, and awareness of these associations should assist the clinical management of people with histories of SARS‐CoV‐2 infection.

**The known:** COVID‐19 has been linked with increased risks of cardiovascular, cerebrovascular, and thrombotic disease events.

**The new:** In our self‐control case series, hospitalisations of people with myocarditis and pericarditis, pulmonary embolism, acute myocardial infarction, and stroke were significantly more frequent after COVID‐19. The increase for acute myocardial infarction was greatest during the week after COVID‐19 onset, and for pulmonary embolism 14–89 days after its onset.

**The implications:** COVID‐19 can impose a considerable respiratory and non‐respiratory morbidity burden beyond the acute period of the disease. Our findings indicate the value of COVID‐19 prevention measures and of the need for ongoing care for people who have had COVID‐19.

The severe acute respiratory syndrome coronavirus 2 (SARS‐CoV‐2), the pathogen of coronavirus disease 2019 (COVID‐19), has caused profound morbidity and mortality around the world. Evidence of its impact beyond the acute illness with respect to its cardiac^1^, ^2^, ^3^, ^4^, ^5^ and neurological effects^2^, ^3^, ^4^, ^6^, ^7^ is increasing, as well as for significant vascular disease events, including thrombosis.^4^, ^8^, ^9^

The estimated risk of myocarditis or pericarditis after SARS‐CoV‐2 infection is 18–21 times as high as for uninfected people.^5^, ^9^ Similarly, the risk of acute myocardial infarction is 3–6 times,^2^, ^3^ that of ischaemic stroke 3–10 times,^2^, ^3^, ^7^ and the risk of venous thrombo‐embolism up to eight times as high.^8^, ^10^, ^11^ These risk differences are greater than for other viral respiratory infections^11^ or for vaccinated people not infected by SARS‐CoV‐2.^5^, ^9^

The COVID‐19 pandemic and our responses have rapidly evolved since SARS‐CoV‐2 was first detected in Victoria on 25 January 2020.^12^ Vaccination programs were progressively rolled out in Australia from 22 February 2021, initially targeting people at particular risk of infection and severe disease, including international border, quarantine, and frontline healthcare workers, aged care and disability services staff and residents, people over 70 years of age and Aboriginal and Torres Strait Islander people over 55 years of age, adults with certain medical conditions, and workers in essential services.^13^

Vaccination can reduce the risk of non‐respiratory complications of SARS‐CoV‐2 infection, including the risks of acute myocardial infarction and ischaemic stroke.^14^ However, understanding the baseline risk of these conditions prior to the widespread rollout of vaccination is important. In this article, we describe our assessment of associations between SARS‐CoV‐2 infection and the incidence of selected respiratory and non‐respiratory conditions in Victoria during 25 January 2020 – 31 May 2021, when the Victorian population was largely vaccine‐naïve.

## Methods

The primary dataset comprised laboratory‐confirmed cases of COVID‐19 notified to the Victorian Department of Health during 25 January 2020 – 31 May 2021 and recorded in the Transmission and Response Epidemiology Victoria (TREVi) database. TREVi is a population‐based surveillance system that captures data on COVID‐19 cases notified by medical practitioners and laboratories,^15^ as required by the *Public Health and Wellbeing Act 2008*.^16^ It contains socio‐demographic, clinical, and risk factor data, including dates of illness onset, specimen collection, and diagnosis. The study cohort comprised people infected with the initial circulating SARS‐CoV‐2 strain (or other low incidence strains, such as Alpha, Kappa and Lambda), prior to the emergence of the Delta and Omicron variants and widespread vaccination against SARS‐CoV‐2 (fewer than 500 000 doses administered by the end of May 2021; about 7.6% population coverage).^17^ The study cohort presumably included only people with first infections; second SARS‐CoV‐2 infections in individuals were first identified in Victoria in May 2021.^18^

Data for the primary cohort were linked to the Victorian Admitted Episodes Dataset (VAED) and Victorian Death Index (VDI). The VAED is an administrative dataset comprising demographic, administrative, and limited clinical data for all patients admitted to public and private Victorian health services. Admissions of people with COVID‐19 (identified in TREVi) were obtained from the VAED for the twelve months preceding and the twelve months following the onset of COVID‐19; the period covered for this purpose was 23 January 2019 (twelve months before onset of first reported COVID‐19 case) to 30 September 2021 (data linkage date). The VDI includes data on all deaths in Victoria registered under the *Births, Deaths and Marriages Registration Act 1996*.^19^ Death records for people with COVID‐19 were sourced from TREVi and the VDI. Record linkages were conducted by the Centre for Victorian Data Linkages at the Victorian Department of Health (Supporting Information, supplementary methods 1).

### Study design

We conducted a self‐controlled case series to assess associations between SARS‐CoV‐2 infection and the incidence of hospitalisation with selected respiratory and non‐respiratory conditions. For people in the primary cohort hospitalised at least once during the twelve months preceding or the twelve months after COVID‐19 onset, we compared the condition‐specific incidence of hospitalisation before and after SARS‐CoV‐2 infection. As each person serves as their own control, all time‐invariant confounding is eliminated by this study design.^20^

### Exposure

The exposure for the self‐controlled case series was defined as laboratory‐confirmed SARS‐CoV‐2 infection. The exposure date was defined as being three days prior to the COVID‐19 onset date recorded in TREVi. When the illness onset date was unknown or the infected person was asymptomatic, the collection date for the first SARS‐CoV‐2‐positive specimen or the COVID‐19 diagnosis date were used as proxies for the exposure date.

Hospital admission episodes were grouped as baseline, clearance, or post‐exposure period admissions. The baseline period comprised days 60–365 before the exposure date, and the clearance period comprised days 0–60 before the exposure date. We excluded admissions during the clearance period from the analysis because hospitalisation (as an inpatient or during convalescence) may influence the risk of SARS‐CoV‐2 exposure. For our primary analysis, the post‐exposure period was from day 3 before illness onset to 89 days after onset (days –3 to 89). For our secondary analyses, a series of non‐overlapping post‐exposure intervals were used: from three days before illness onset to six days from onset (days –3 to 6), 7–13 days, 14–29 days, 30–59 days, 60–89 days, 90–182 days, and 183–365 days (Box 1).

Box 1 Schematic depiction of the self‐controlled case series study design*

**Figure:**
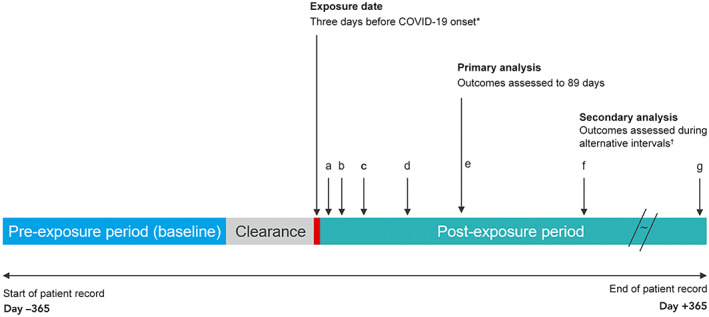
COVID‐19 = coronavirus disease 2019; SARS‐CoV‐2 = severe acute respiratory syndrome coronavirus 2. * For asymptomatic infections: specimen collection date for the first SARS‐CoV‐2‐positive specimen or the COVID‐19 diagnosis date. † Time periods for the sensitivity analyses (with respect to illness onset): a. –3 to 6 days; b. 7–13 days; c. 14–29 days; d. 30–59 days; e. 60–89 days; f. 90–182 days; g. 183–365 days.

### COVID‐19‐related hospitalisation

People with laboratory‐confirmed COVID‐19 were classified as being hospitalised because of or with acute COVID‐19 if this was recorded in TREVi (obtained by patient, clinician, or next‐of‐kin interview), if they were identified as being hospitalised using other enhanced surveillance mechanisms,^21^ or if record linkage with VAED identified a COVID‐19‐related hospitalisation (International Classification of Diseases, tenth revision, Australian modification [ICD‐10‐AM] diagnoses codes; Supporting Information, supplementary methods 2).

### Association between SARS‐CoV‐2 infection and selected respiratory and non‐respiratory outcomes

We assessed whether each person with notified COVID‐19 in the self‐controlled case series had had at least one acute hospital admission because of cardiac events (acute myocardial infarction, ischaemic heart disease, myocarditis or pericarditis), atrial fibrillation, cerebrovascular events (cerebral infarction and non‐ischaemic stroke, intracranial haemorrhage, occlusion and stenosis, subarachnoid haemorrhage, transitory cerebral ischaemic attack), other arterial embolic events, venous thrombo‐embolism (pulmonary embolism, lower limb embolism, phlebitis, thrombophlebitis, splanchnic or other venous thrombosis), thrombocytopenia and coagulation disorders, encephalitis, myelitis and encephalomyelitis, bleeding events and anaemias, acute kidney failure, or respiratory events (infectious, non‐infectious, asthma). Injury and urinary tract infection were explored as negative control outcomes. All outcomes were identified by ICD‐10‐AM codes in linked VAED records (Supporting Information, table 1). Time of outcome was categorised relative to the person's exposure date. We included only the first admitted episode for each person, condition, and exposure period (baseline, post‐exposure).

In our primary self‐controlled case series analysis, an outcome was deemed to have occurred if coded in any of the 40 VAED diagnosis fields, the admission date was later than the exposure date, and the discharge date was less than 90 days after COVID‐19 onset. In secondary analyses, an outcome was deemed to have occurred if coded as the first (principal) diagnosis and the admission date was within the time intervals defined above; people admitted more than once with the same condition were included only once in our analyses (in the interval closest to COVID‐19 onset) (Supporting Information, supplementary methods 2).

### Statistical analyses

The hospitalisation rate for people with COVID‐19, reasons for hospitalisation, and the socio‐demographic and clinical characteristics of people with acute COVID‐19 are reported as descriptive statistics.

For the self‐controlled case series, the incidence of each outcome was estimated for the baseline and post‐exposure periods, censored at 365 days or the day after an outcome. To compare the incidence of outcomes during the post‐exposure and baseline periods, we calculated incidence rate ratios (IRRs) with 95% confidence intervals (CIs) in a conditional probability Poisson fixed effects regression model with robust standard errors. Analyses were performed in Stata 16.0; graphs were generated in R 4.0.2 (R Foundation for Statistical Computing).

### Ethics approval

Our study was approved by the Victorian Department of Health Research Ethics Committee (LNR/76271/DHHS‐2021‐272262(v2)) and registered with the Monash University Human Research Ethics Committee (30071).

## Results

During 25 January 2020 – 31 May 2021, 20 594 COVID‐19 cases were notified in Victoria. A total of 2494 people (12.1%) were recorded in TREVi as having been hospitalised with COVID‐19; linkage with VAED identified an additional 492 people with ICD‐10‐AM‐coded COVID‐19‐related hospitalisations and six without such coding but determined to have acquired SARS‐CoV‐2 infections in hospital (as identified in a separate study^22^), yielding a total of 2992 people hospitalised because of or with acute COVID‐19 (14.5% of notified cases; Box 2, Box 3).

Box 2 **Study flowchart, showing total number of laboratory‐confirmed COVID‐19 cases**

**Figure:**
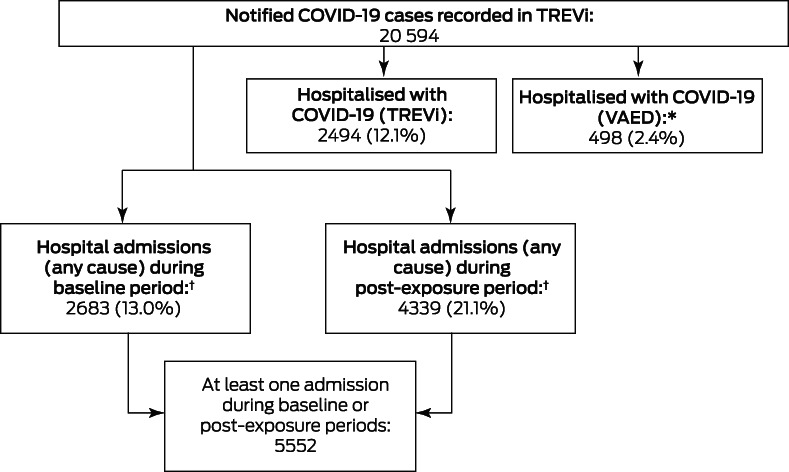
COVID‐19 = coronavirus disease 2019; SARS‐CoV‐2 = severe acute respiratory syndrome coronavirus 2. * Includes six cases of hospital‐acquired SARS‐CoV‐2 infection not coded as such (identified in a separate study^22^). † Includes 1470 people with hospital admissions during both the baseline and post‐exposure periods.

Box 3 **Characteristics of people with notified COVID‐19, Victoria, 25 January 2020 – 31 May 2021, by hospitalisation status**

**Table:** 

	Hospitalised	Not hospitalised	Total
Number of people	2992	17 602	20 594
Age group (years)			
0–4	40 (6.8%)	550 (93.2%)	590 [2.9%]
5–14	659 (6.0%)	10 244 (94.0%)	10 903 [52.9%]
15–44	677 (15.5%)	3684 (84.5%)	4361 [21.2%]
45–64	43 (2.9%)	1437 (97.1%)	1480 [7.2%]
65–79	592 (41.5%)	836 (58.5%)	1428 [6.9%]
80 or older	981 (53.5%)	851 (46.5%)	1832 [8.9%]
Sex			
Male	1350 (13.7%)	8499 (86.3%)	9849 [47.8%]
Female	1640 (15.3%)	9094 (84.7%)	10 734 [52.1%]
Other	2 (25%)	6 (75%)	8 [< 0.1%]
Missing data	0	3 (100%)	3 [< 0.1%]
Indigenous status			
Aboriginal or Torres Strait Islander	2920 (14.7%)	17 002 (85.3%)	19 922 [96.7%]
Non‐Indigenous	22 (21.0%)	83 (79.0%)	105 [0.5%]
Missing data	50 (8.8%)	517 (91.2%)	567 [2.8%]
Birth country			
Australia	1283 (15.2%)	7149 (84.8%)	8432 [40.9%]
Other	1618 (15.4%)	8858 (84.6%)	10 476 [50.9%]
Missing data	91 (5.4%)	1595 (94.6%)	1686 [8.2%]
Culturally and linguistically diverse*			
Yes	1182 (12.5%)	8310 (87.5%)	9492 [46.1%]
No	637 (11.4%)	4936 (88.6%)	5573 [27.1%]
Missing data	1173 (21.2%)	4356 (78.8%)	5529 [26.8%]
Interpreter required			
Yes	424 (20.8%)	1613 (79.2%)	2037 [9.9%]
No	2316 (13.5%)	14 793 (86.5%)	17 109 [83.1%]
Missing data	252 (17.4%)	1196 (82.6%)	1448 [7.0%]
Residential location^†^			
Metropolitan	2791 (14.8%)	16 116 (85.2%)	18 907 [91.8%]
Regional	180 (12.6%)	1250 (87.4%)	1430 [6.9%]
Missing data	21 (8.2%)	236 (91.8%)	257 [1.2%]
Residential socio‐economic status (quintiles)^‡^			
1 (highest disadvantage)	328 (12.7%)	2262 (87.3%)	2590 [12.6%]
2	200 (10.9%)	1643 (89.1%)	1843 [8.9%]
3	64 (17%)	310 (82.9%)	374 [1.8%]
4	1602 (15.3%)	8878 (84.7%)	10 480 [50.9%]
5 (lowest disadvantage)	777 (15.4%)	4273 (84.6%)	5050 [24.5%]
Missing data	21 (8.2%)	236 (91.8%)	257 [1.2%]
Symptoms at testing			
Symptomatic	2104 (13.8%)	13 089 (86.2%)	15 193 [73.8%]
Asymptomatic	885 (16.6%)	4451 (83.4%)	5336 [25.9%]
Missing data	3 (5%)	62 (95%)	65 [0.3%]
Died			
Yes	559 (68.2%)	261 (31.8%)	820 [4.0%]
No	2432 (12.3%)	17 335 (87.7%)	19 767 [96.0%]
Missing data	1 (14%)	6 (85.7%)	7 [< 0.1%]

* People born in predominantly non‐English speaking countries (ie, other than Australia, New Zealand, Ireland, South Africa, United States of America, Canada, United Kingdom) or who speak a language other than English at home.

† The residential address is geocoded according to a managed set of corporate reference data maintained by the Victorian Department of Health (Health Reference Data).^15^, ^23^

‡ Socio‐Economic Indexes for Areas Index of Relative Socio‐economic Disadvantage (IRSD).^24^

A total of 9336 hospital admissions (any cause) within twelve months of COVID‐19 onset were recorded for 4339 people (21.1% of notified COVID‐19 cases), including 2334 within twelve days of COVID‐19 onset (25% of all post‐exposure admissions) and 4668 within 115 days (50%). The median number of admissions per person was one (interquartile range, 1–2; range, 1–255). Fewer than ten people required more than 50 admissions, most for the treatment of chronic kidney disease. Apart from kidney disease or cancer treatment, the most frequent reasons for admission during the post‐exposure period were respiratory symptoms or diagnoses typical for COVID‐19, including viral pneumonia (780 admissions, 8.4%), unspecified coronavirus infection (358, 3.9%), cough (234, 2.5%), fever (192, 2.1%), and dyspnoea (178, 1.9%) (Supporting Information, table 2).

### Incidence of hospitalisation: post‐exposure period (to 89 days) *v* baseline period

The incidence of hospitalisation with myocarditis and pericarditis (IRR, 14.8; 95% CI, 3.2–68.3), thrombocytopenia (IRR, 7.4; 95% CI, 4.4–12.5), coagulative disorders (IRR, 4.6; 95% CI, 1.5–14.5), acute kidney failure (IRR, 4.5; 95% CI, 3.8–5.3), acute myocardial infarction (IRR, 3.9; 95% CI, 2.6–5.8), and cerebral infarction (IRR, 2.3; 95% CI, 1.4–3.9) were each higher during the post‐exposure period than during the baseline period, as were those of venous thrombo‐embolic events (pulmonary embolism: IRR, 6.4; 95% CI, 3.6–11.4; lower limb embolism, phlebitis and thrombophlebitis: IRR, 5.7; 95% CI, 2.8–11.7) (Box 4).

Box 4 Hospitalisations of people with laboratory‐confirmed COVID‐19 during the baseline and post‐exposure periods (to 89 days) and incidence rate ratios (IRRs) with 95% confidence intervals (CIs) (post‐exposure *v* baseline)*

**Figure:**
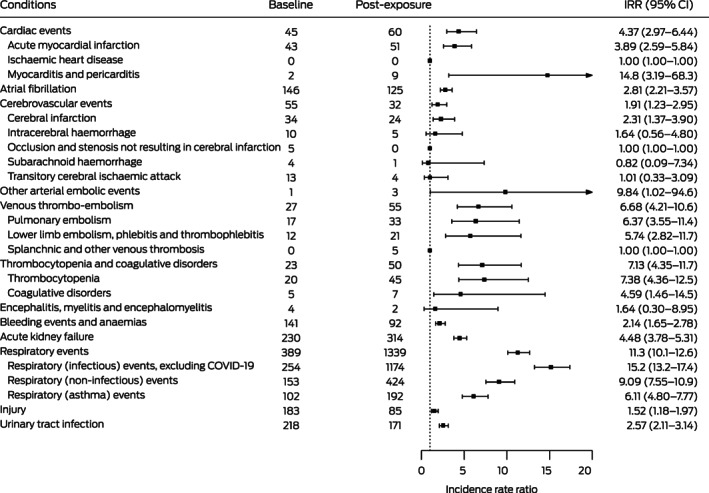
COVID‐19 = coronavirus disease 2019. * Baseline: days 365–60 before exposure (severe acute respiratory syndrome coronavirus 2 infection; ie, 305 days); post‐exposure: date of exposure (three days before COVID‐19 onset) to 89 days after COVID‐19 onset (ie, 93 days). † Includes two cases of pericarditis, one during the baseline period and one during post‐exposure period (day 4 after COVID‐19 onset).

Five people had embolisms or thrombosis of other specified veins (including splanchnic vessels) during the post‐exposure period, but the absence of baseline events precluded IRR estimation. All but one of these patients was admitted to hospital at least 14 days after COVID‐19 onset (range, 4–66 days), suggesting delays in the onset or diagnosis of such events. Twenty‐four people had cerebral infarctions within 89 days of COVID‐19 onset, none specifically coded as cerebral infarction caused by central venous or portal vein thrombosis.

The rate of hospitalisation with respiratory conditions was markedly higher post‐exposure than the baseline period, including infectious respiratory events (IRR, 15.2; 95% CI, 13.2–17.4), non‐infectious respiratory events (IRR, 9.1; 95% CI, 7.6–10.9), and asthma events (IRR, 6.1; 95% CI, 4.8–7.8). Finally, the rate of hospitalisation with control conditions (injuries, urinary tract infections) were also higher during the post‐exposure period than the baseline period (Box 4).

### Secondary analyses

The incidence of hospitalisation with acute myocardial infarction (three cases; IRR, 5.1; 95% CI, 1.5–17) or intracerebral haemorrhage (two cases; IRR, 10; 95% CI, 2.1–50) were each higher during days –3 to 6 than during the baseline period. The incidence rate ratio for venous thrombo‐embolic events was highest 14–29 days after COVID‐19 onset (four cases; IRR, 5.5; 95% CI, 1.8–17) and 30–89 days after onset (total of twelve cases; IRR, 4.4; 95% CI, 1.7–11). The incidence rate ratio for respiratory symptom‐related hospitalisations was highest up to 30 days after COVID‐19 onset (days –3 to 6: 621 cases; IRR, 89 [95% CI, 76.4–104]; days 7–13: 326 cases; IRR, 67.0 [95% CI, 56.4–79.7]; days 14 to 29: 64 cases; IRR, 5.8 [95% CI, 4.4–7.6]) (Supporting Information, table 3).

## Discussion

We found that SARS‐CoV‐2 infection was associated with higher incidence of hospitalisation with several non‐respiratory conditions in Victoria during January 2020 – May 2021, particularly myocarditis and pericarditis, pulmonary embolism and other venous thrombo‐embolisms, thrombocytopenia, and coagulative disorders; weaker associations with hospitalisation with acute kidney failure, acute myocardial infarction, and cerebral infarction were also found.

The incidence of hospitalisation following venous thrombo‐embolic events was markedly higher than baseline within 90 days after COVID‐19 onset, consistent with other studies;^8^, ^10^ the increase in incidence was greatest 14–59 days after COVID‐19 onset. Pulmonary embolism is a recognised complication of SARS‐CoV‐2 infection, but its non‐specific symptoms and sometimes late onset and diagnosis can delay treatment. In our study, most cases were diagnosed after the patient had been discharged from the COVID‐19‐related admission. A low threshold for investigation is therefore warranted in people with COVID‐19 or history of past SARS‐CoV‐2 infection.

The incidence of hospitalisation with acute myocardial infarction was higher after than before SARS‐CoV‐2 infection (IRR, 3.9; 95% CI, 2.6–5.8); 51 people had acute myocardial infarctions within 90 days of COVID‐19 onset. The increase in incidence was greatest between the presumptive infection date and six days after COVID‐19 onset (three cases; IRR, 5.1; 95% CI, 1.5–17), consistent with the findings of other studies.^2^

The incidence of hospitalisation with cerebral infarction was twice as high after COVID‐19 onset as during the baseline period. Other investigators have estimated the risk of stroke to be 2–13 times as high for people with COVID‐19.^2^, ^3^, ^7^ The pathophysiology of cerebrovascular involvement is unclear, but may be related to substantial endothelial disruption in highly vascularised organs such as the brain.^25^

Complications, including cardiac and thrombo‐embolic events, are important features of COVID‐19 as a multi‐organ disease. The increases in hospitalisation rates we found were statistically and clinically significant, despite the often small numbers. We examined the initial sixteen months of the COVID‐19 pandemic in Victoria, before widespread vaccination and the larger Delta and Omicron SARS‐CoV‐2 epidemic waves. The risk of complications may have risen with the increase in COVID‐19 incidence since the period of our study, but whether population vaccination has modified risk is unclear, as is the association of specific SARS‐CoV‐2 variants with particular complications. Our findings provide a useful baseline for exploring variations in complication rates modified by these factors.

Some COVID‐19 complications clinically resemble those reported after vaccination against SARS‐CoV‐2, which is important when evaluating putatively post‐vaccination adverse events. Further, we found that the incidence of hospitalisation with severe cardiac and thrombo‐embolic events after SARS‐CoV‐2 infection was higher than the reported risk of these events after vaccination.^5^, ^8^, ^9^

During the period covered by our study, case ascertainment in Victoria was high and the incidence of COVID‐19 relatively low to moderate (6.5–549 cases per 100 000 population^12^). Further, enhanced hospital‐based surveillance,^21^ expanded diagnostic laboratory services, and a health system not overwhelmed by the pandemic facilitated ascertainment of hospitalisations of all people with COVID‐19 in Victoria, enabling analysis at the population level over an extended period, superior to a single site or short period study. Analysing linked population datasets facilitated both specific exposure and outcomes measures. Further, the self‐controlled case series study design controlled for time‐invariant confounders such as socio‐demographic factors, comorbid conditions, and other risk factors, in contrast to studies with a general population or matched control group as comparators^11^, ^26^, ^27^ and therefore subject to uncontrolled confounding.

### Limitations

First, we analysed surveillance data based on notifications to the Victorian Department of Health; given that about one‐quarter of included SARS‐CoV‐2 infections were asymptomatic, some SARS‐CoV‐2‐infected people may not have been reported and therefore not included in our analysis. Second, we assessed outcomes only for people with notified COVID‐19 who were admitted to hospital. Third, medical conditions leading to hospitalisation could have arisen at any time prior to or during hospital admission, including admissions with COVID‐19; we therefore included all admissions commencing within 90 days of COVID‐19 onset and excluded admissions lasting 90 days or longer.

Fourth, diagnostic coding errors may have led to misclassification, but local audits have found that VAED coding quality is high.^28^ Coding accuracy can vary by condition; general or non‐specific diagnoses (eg, bleeding) may be under‐reported in administrative datasets,^29^ but events such as pulmonary embolism, stroke, and acute myocardial infarction are more reliably coded.^30^ Fifth, we did not account for time‐varying confounding by factors such as age. Changes in lifestyle or other factors may have moderated risk for specific conditions, such as acute myocardial infarction and stroke, and such changes may have been more pronounced during periods of restrictions limiting movement and social interactions. However, major changes in risk across the study period are unlikely.

Sixth, the incidence of the two negative control conditions was also increased during the post‐exposure period. This may reflect genuine increases in the risks of, for example, falls during COVID‐19 convalescence or catheter‐related urinary tract infections during hospitalisation with COVID‐19, or an unidentified source of bias or confounding. Reverse causality is also possible; that is, the outcome of interest (eg, an injury leading to hospitalisation) preceded the exposure (SARS‐CoV‐2 infection), and the person was tested for COVID‐19 only after admission. Cases in which people were admitted to hospital during the three days between infection and COVID‐19 onset could be a source of such bias. However, outcomes during this period could be genuinely related to systemic responses to SARS‐CoV‐2 infection. Another investigation that examined this source of bias in a self‐controlled case series found that the risk of significant cardiac and cerebrovascular events after infection was elevated whether day 0 patients were included or not.^3^ The results of a sensitivity analysis excluding outcomes during days –3 to 0 were similar to those of our main analysis (data not shown).

### Conclusion

Our findings are consistent with other reports regarding non‐respiratory conditions after SARS‐CoV‐2 infection. These findings — coupled with growing knowledge of the post‐COVID‐19 condition (“long COVID”), defined as the presence of symptoms lasting at least two months in a person with a history of SARS‐CoV‐2 infection and not explained by an alternative diagnosis, usually three months after COVID‐19 onset^31^ — highlight the continuing clinical, public health, and societal challenges for managing COVID‐19. We did not include the post‐COVID‐19 condition in our study because hospital dataset coding classification standards for recording it have only recently been introduced; further, it does not usually lead to hospitalisation.

Our findings indicate the need for ongoing COVID‐19 mitigation measures, including vaccination, and support the early diagnosis and management of complications in people with histories of SARS‐CoV‐2 infection. The pathophysiological mechanisms underlying symptom persistence and the development of major complications still need to be elucidated, the prevalence of the post‐COVID‐19 condition (by vaccination status) established, and the risks of complications following vaccination quantified.

## Open access

Open access publishing facilitated by Monash University, as part of the Wiley – Monash University agreement via the Council of Australian University Librarians.

## Competing interests

No relevant disclosures.

## Supporting information


**Table S1** ICD‐10‐AM codes used to assess outcomes of interest
**Table S2**. Twenty most frequent reasons for hospitalisation of people with COVID‐19 (principal [first] diagnosis), Victoria, 25 January 2020 – 31 May 2021
**Table S3**. Secondary analyses: Incidence of hospitalisation during baseline and various post‐exposure periods:* incidence rate ratios (IRRs) with 95% confidence intervals (CIs)
