# Supplementary material for: Associations between COVID‐19 and hospitalisation with respiratory and non‐respiratory conditions: a record linkage study
Source: Med J Aust. 2022 Nov 14;218(1):33–9. doi: 10.5694/mja2.51778 (PMC10100490; doi:10.5694/mja2.51778)
Supplement: Supplementary file 1 — Table S1 ICD‐10‐AM codes used to assess outcomes of interest Table S2. Twenty most frequent reasons for hospitalisation of people with COVID‐19 (principal [first] diagnosis), Victoria, 25 January 2020 – 31 May 2021 Table S3. Secondary analyses: Incidence of hospitalisation during baseline and various post‐exposure periods:* incidence rate ratios (IRRs) with 95% confidence intervals (CIs) [file MJA2-218-33-s001.pdf]

The Medical Journal of Australia

Supporting Information

Supplementary methods and results

This appendix was part of the submitted manuscript and has been peer reviewed.
It is posted as supplied by the authors.

Appendix to: Rowe SL, Leder K, Dyson K, et al. Associations between COVID-19 and hospitalisation with respiratory and non-respiratory conditions: a record linkage study. *Med J Aust* 2023; doi: 10.5694/mja2.51778.

Supplementary methods

1. Data sources and linkage

Transmission and Response Epidemiology Victoria

The Transmission and Response Epidemiology Victoria (TREVi) database is a population-based surveillance system that captures cases of COVID-19 notified by medical practitioners and laboratories under the Public Health and Wellbeing Act 2008.(1) It contains socio-demographic, clinical and risk factor data, including hospital and /or intensive care unit admissions occurring during the period of illness, dates of onset of illness, specimen collection and diagnosis.(2)

Victorian Admitted Episode Dataset

The Victorian Admitted Episodes Dataset (VAED) is an administrative dataset that contains demographic, administrative and clinical data relating to the causes, effects, and nature of illness occurring among patients admitted to Victorian health services. It contains reasons for admission and procedures performed but excludes data on treatments administered during hospitalisation. All Victorian public and private hospitals, including rehabilitation centres, extended care facilities and day procedure centres, are required to report a minimum dataset for each admitted patient episode.(3)

Victorian Death Index

The Victorian Death Index (VDI) captures all registrable deaths occurring in Victoria under the Births, Deaths and Marriages Registration Act 1996.(4) Funeral directors organising the disposal of a deceased person and medical practitioners certifying the death are required to register the death. Information captured includes identifying details of the deceased, cause of death, date of death and place of death.

Data linkage

Record linkages were carried out by the Centre for Victorian Data Linkages (CVDL) at the Victorian Government Department of Health. CVLD is a specialist data linkage unit established in 2009 with funding from the national Population Health Research Network (PHRN) and the Victorian Government. The CVDL is an accredited Commonwealth Integrating Authority, which means that integration of high risks projects involving the Commonwealth data for statistical and research purposes is undertaken safely and securely.(5)

For this study, there were several discrete steps involved in the linkage process. First, data cleaning and standardisation steps were performed. For example, variables are standardised (first name and middle name parsed; variable names renamed to a consistent nomenclature; variable values consistently formatted; cleaning of invalid entries and coding consistently for missing data/blanks). Second, enrichment of VAED with the Better Patient Data (BPD) dataset was carried out. BPD is a file that contains patient names and Medicare numbers and is used to supplement VAED with patient name and to provide enhanced capacity to link the dataset with others such as TREVi. (Medicare is a universal healthcare scheme providing free or subsidised medical care to Australian residents. A Medicare number is provided to all eligible residents; and whilst the numbers is regarded as non-unique, it is commonly available in administrative healthcare datasets and is considered a suitable proxy for unique healthcare identifier). Third, a spine-based record linkage algorithm links “events” (for example, admissions, tests etc.) to an “entity” (a unique person). The linkage algorithm takes a deterministic approach using first name, last name and date of birth. Fourth, linkage keys generated from the creation of the spine enabled the content from each data source to be brought together to support analysis.

2. Hospitalisations data

There were three distinct uses of VAED codes to address three differing objectives throughout the study. As such, assessment of VAED codes varied, as described below.

Assessment of COVID-19-related hospitalisation of people with notified COVID-19

The first part of the descriptive analysis described the characteristics of the primary cohort (people with COVID-19) including rate of hospitalisation because of acute COVID-19 illness.

A person's requirement for hospitalisation because of and during acute COVID-19 was sourced from TREVi or VAED.

TREVI: As part of routine public health investigation for COVID-19 cases, a person's acuity is assessed by patient, clinician or next-of-kin interview and recorded in TREVi. During the pandemic, TREVi data were enhanced by a COVID-19 patient monitoring surveillance system, established in April 2020. This system involved transmission of daily data from Victorian health services to the Victorian Department of Health, sourced from infection control practitioners at each service (6).

VAED: COVID-19 cases were linked with VAED to enable the identification of COVID-19-related hospitalisations not previously captured in TREVi. COVID-19 cases with one or more of the following codes captured on *any of 40 diagnoses (Principal* or other diagnoses) during their admitted episode were considered hospitalised because of or with COVID-19.

- U071: Emergency use of U071 [COVID-19, virus identified];
- U072: Emergency use of U071 [COVID-19, virus not identified];
- B972: Coronavirus as the cause of disease classified to other chapters;
- B342: Coronavirus infection, unspecified site.

Our definition included diagnoses identified as *Primary* or *Complicating conditions,* as reflected in the condition onset flag. This is in line with guidance provided by the Australian Classification Exchange coding rules relating to the classification of COVID-19: Coding Rule – Coronavirus disease 2019 (COVID-19) (effective 1 January 2020; updated 27 March 2020) Ref No: TN1530.(7).

As part of the assessment, the *first* COVID-19-related admitted episode identified subsequent to the patient's COVID-19 illness onset was contributed to this summary statistic.

Assessment of any reason for hospitalisation among notified COVID-19 cases

The second part of the descriptive analysis assessed the most frequent *reasons* for hospitalisation among the primary cohort of COVID-19 patients with one or more *all-cause* linked admitted episodes during days -3 to 365 days relative to COVID-19 illness onset.

To identify reason for admission, only the *principal (first)* diagnosis codes were assessed. In the VAED, the *principal* diagnosis reflects the condition "chiefly responsible for occasioning an episode of admitted patient care…" (3).

As part of the assessment, all admissions were summarised, and patients with more than one admitted episode therefore contributed to these summary statistics.

Assessment of respiratory and non-respiratory outcomes among people with notified COVID-19 as part of the self-controlled case series

The main objective of the study was to explore associations between SARS-CoV-2 infection and selected respiratory and non-respiratory outcomes (Table 1). The analysis comprised a primary analysis and a series of secondary analyses.

In the primary analyses, a condition was classified as being present in the post-exposure period if:

- it was coded as such in *any* of *40 diagnoses (Principal, Primary or Complication)* in VAED; AND
- if the admission occurred between -3 and <90 days of COVID-19 illness onset; AND
- if the discharge date also occurred <90 days of illness onset.

This more inclusive approach, including all 40 diagnosis fields, is consistent with Australian coding classification standards, which prescribe that the principal reason for admission to hospital be coded in the first (principal) diagnosis code (e.g. cough, or unspecified acute lower respiratory infection), with other conditions suspected at the time of admission – and subsequently confirmed during the current episode of admitted patient care (e.g. pneumonia, acute myocardial infarction, stroke, unstable angina) – assigned as other primary diagnoses captured in one of the remaining 40 diagnoses fields. Further, and as we sought

to explore complications of SARS-CoV-2 infection, codes with a Complication prefix captured across the possible 40 diagnosis fields were also included.(8)

Only the first admitted episode per person, per exposure period (baseline or post-SARS-CoV-2 infection), per condition was included in the analyses. For example, if a case had two admissions coded as cerebral infarction in the baseline period, and two subsequent admissions coded as cerebral infarction in the post-exposure period, only two of these four admitted episodes would be included in the analysis: the first to occur in the pre-exposure period, and the first to occur in the post exposure period. If the same case also had admitted episodes for another condition of interest, e.g. acute kidney failure, the first of these would also be included in the analysis and contribute data to the assessment of risk of acute kidney failure.

In the secondary analysis, we assessed each outcome of interest in non-overlapping time increments as specified below using the same diagnoses codes as the primary analyses, however, only *Principal (first)* diagnoses code was assessed.

a) day -3 to 6;
b) day 7 to 13;
c) day 14 to 29;
d) day 30 to 59;
e) day 60 to 89;
f) day 90 to 182; and
g) day 183 to 365.

As with the primary analysis, only the first admitted episode, per person, per condition was included in the secondary analysis. As such, among cases with more than one admitted episode occurring for the same condition of interest in the post-exposure period, only the first admitted episode in the time interval most proximal to their COVID-19 illness onset was included in the analysis. For example, if a case had an admitted episode for acute myocardial infarction in time-point (a); and a second admitted episode with acute myocardial infarction in time-point (d), only the admission identified in time-point (a) was included in the analysis.

Table 1. ICD-10-AM codes used to assess outcomes of interest

Condition	Code	Description
**Cardiac events**		All codes below
**Acute myocardial infarction**	I21	Acute myocardial infarction
**Ischaemic heart disease**	I20	Other acute ischaemic heart diseases*
	I256	Silent myocardial ischaemia
**Myocarditis and pericarditis**	I40	Acute myocarditis
	I41	Myocarditis in diseases classified elsewhere
	I514	Myocarditis, unspecified
	I30	Acute pericarditis
	B322	Viral carditis
**Cerebrovascular events**		All codes below
**Cerebral infarction†**	I63	Cerebral infarction
	I64	Stroke, not specified as haemorrhage or infarction
**Intracerebral haemorrhage**	I61	Intracerebral haemorrhage
	I62	Other nontraumatic intracranial haemorrhage
**Occlusion and stenosis not resulting in cerebral infarction**	I65	Occlusion and stenosis of precerebral arteries, not resulting in cerebral infarction
	I66	Occlusion and stenosis of cerebral arteries, not resulting in cerebral infarction
**Subarachnoid haemorrhage**	I60	Subarachnoid haemorrhage
**Transitory cerebral ischemic attack**	G45	Transient cerebral ischaemic attacks and related syndromes
		Includes
	G450	Vertebro-basilar artery syndrome
	G451	Carotid artery syndrome (hemispheric)
	G452	Multiple and bilateral precerebral artery syndromes
	G453	Amaurosis fugax
	G454	Transient global amnesia
	G458	Other transient cerebral ischaemic attacks and related syndromes
	G459	Transient cerebral ischaemic attack, unspecified
**Other arterial embolic events**	I740	Embolism and thrombosis of abdominal aorta
	I741	Embolism and thrombosis of other and unspecified parts of aorta
	I742	Embolism and thrombosis of arteries of upper extremities
	I744	Embolism and thrombosis of arteries of extremities, unspecified
	I745	Embolism and thrombosis of iliac artery
	I748	Embolism and thrombosis of other arteries
	I749	Embolism and thrombosis of unspecified artery
**Venous thromboembolic events**		All codes below
**Pulmonary Embolism**	I26	Pulmonary embolism
**Lower limb embolism, phlebitis and thrombophlebitis (superficial vessels, deep vessels, other lower extremities unspecified)**	I800	Phlebitis and thrombophlebitis of superficial vessels of lower extremities
	I801	Phlebitis and thrombophlebitis of femoral vein
	I802	Phlebitis and thrombophlebitis of other deep vessels of lower extremities
	I803	Phlebitis and thrombophlebitis of lower extremities, unspecified
	I743	Embolism and thrombosis of arteries of lower extremities
**Splanchnic and other venous thrombosis**	1809	Phlebitis and thrombophlebitis of unspecified site
	I81	Portal vein thrombosis
	I822	Embolism or thrombosis of vena cava
	I828	Embolism and thrombosis of other specified veins
	I829	Embolism and thrombosis of unspecified vein
	I829	Embolism or thrombosis of renal vein

Condition	Code	Description
**Thrombocytopenia and coagulative disorders**		All codes below
**Thrombocytopenia**	D693	Idiopathic thrombocytopenic purpura
	D694	Other primary thrombocytopenia
	D695	Secondary thrombocytopenia
	D696	Thrombocytopenia, unspecified
**Coagulative disorders**	D65	Disseminated intravascular coagulation
	D688	Other specified coagulation defects
	D689	Coagulation defect unspecified
**Encephalitis, myelitis and encephalomyelitis**	G040	Acute disseminated encephalitis
	G048	Other encephalitis, myelitis and encephalomyelitis
	G049	Encephalitis, myelitis and encephalomyelitis, unspecified
	G05	Encephalitis, myelitis and encephalomyelitis in diseases classified elsewhere
**Bleeding events and anaemias**	D62	Acute post-haemorrhagic anaemia
	K922	Gastrointestinal haemorrhage, unspecified
	R04	Haemorrhage from respiratory passages
	R31	Unspecified haematuria
	R58	Haemorrhage, not elsewhere classified
**Acute kidney failure**	N17	Acute kidney failure
	N19	Unspecified kidney failure
**Respiratory events**		All codes below
**Respiratory (infectious) events^**	J00–J06	Acute upper respiratory infections
	J09–J18	Influenza and pneumonia
	J20–J22	Other acute lower respiratory infections
**Respiratory (non-infectious) events**	J30–J39	Other diseases of upper respiratory tract
	J40	Bronchitis, not specified as acute or chronic
	J41	Simple and mucopurulent chronic bronchitis
	J42	Unspecified chronic bronchitis
	J43	Emphysema
	J47	Bronchiectasis
	J60–J70	Lung diseases due to external agents; respiratory conditions due to other external agents
	J80–J84	Other respiratory diseases principally affecting the interstitium
	J85–J86	Suppurative and necrotic conditions of lower respiratory tract
	J90–J94	Other diseases of pleura
	J95–J99	Other diseases of the respiratory system
**Respiratory (asthma) events**	J44	Other chronic obstructive pulmonary disease
	J45	Asthma
	J46	Status asthmaticus
**Atrial fibrillation**	I480	Paroxysmal atrial fibrillation
	I483	Typical atrial flutter
	I484	Atypical atrial flutter
	I489	Atrial fibrillation and atrial flutter, unspecified
	I49	Other cardiac arrhythmias
**Negative control**		
**Injury**	S00-S09	Injuries to the head
**Urinary tract infection**	N390	Urinary tract infection, site not specified

* includes coronary thrombosis not resulting in myocardial infarction;

† includes I636 cerebral infarction due to cerebral venous thrombosis, non-pyogenic;

^ excludes records where COVID-19 diagnoses codes are also used, U071: emergency use of U071 [COVID-19, virus identified]; U072: emergency use of U071 [COVID-19, virus not identified]; B972: coronavirus as the cause of disease classified to other chapters; B342: coronavirus infection, unspecified site.

Supplementary results

Table 2. Twenty most frequent reasons for hospitalisation of people with COVID-19 (principal [first] diagnosis), Victoria, 25 January 2020 – 31 May 2021

Principal diagnosis*	Number	Proportion
Total number of principal (first) diagnoses†	9336	
Other viral pneumonia	780	8.4%
Coronavirus infection unspecified site	358	3.9%
Cough	234	2.5%
Fever unspecified	192	2.1%
Dyspnoea	178	1.9%
Chest pain unspecified	130	1.4%
Iron deficiency anaemia	126	1.4%
Pneumonia unspecified	89	1.0%
Malaise and fatigue	82	0.9%
Other and unspecified abdominal pain	73	0.8%
Congestive heart failure	69	0.7%
Viral pneumonia unspecified	59	0.6%
Headache	54	0.6%
Other chest pain	51	0.6%
Acute lower respiratory infection (unspecified)	51	0.6%
Pain localised to upper abdomen	48	0.5%
Gastrointestinal haemorrhage (unspecified)	45	0.5%
Respiratory failure (unspecified type I)	43	0.5%
Asphyxia	41	0.4%
Atrial fibrillation and flutter (unspecified)	41	0.4%

***** excludes the following diagnoses: extracorporeal dialysis; pharmacotherapy session for neoplasm, single spontaneous delivery, cataract unspecified, single delivery by caesarean section, hyperemesis gravidarum, in vitro fertilisation, transfer from residential aged care service.

† multiple admissions per person included.

Table 3. Secondary analyses: Incidence of hospitalisation during baseline and various post-exposure periods:* incidence rate ratios (IRRs) with 95% confidence intervals (CIs)

		Period		
	**Condition**	**Baseline**	**Post-exposure**	**IRR (95% CI)**
Cardiac events	**Arterial cardiac events (summary)**	19	-	1
	-3 to <7		3	**4.82 (1.43-16.3)**
	7 to <14		0	nc
	14 to <30		1	1.00 (0.13-7.49)
	30 to <60		2	1.07 (0.25-4.59)
	60- to <90		0	nc
	90 to <183		8	1.38 (0.60-3.15)
	183 to <366		5	0.44 (0.16-1.17)
	Acute myocardial infarction	18	-	1
	-3 to <7		3	**5.08 (1.50-17.3)**
	7 to <14		0	nc
	14 to <30		0	nc
	30 to <60		2	1.13 (0.26-4.87)
	60- to <90		0	nc
	90 to <183		7	1.28 (0.53-3.05)
	183 to <366		5	0.46 (0.17-1.25)
	Myocarditis and pericarditis	1		1
	-3 to <7		0	nc
	7 to <14		0	nc
	14 to <30		1	**19.1 (1.2-305)**
	30 to <60		0	nc
	60- to <90		0	nc
	90 to <183		1	3.28 (0.21-52.4)
Cerebrovascular events	**Cerebrovascular events (summary)**	44	-	1
	-3 to <7		5	**3.47 (1.37-8.74)**
	7 to <14		1	0.99 (0.14-7.19)
	14 to <30		2	0.87 (0.21-3.57)
	30 to <60		8	1.85 (0.87-3.93)
	60- to <90		4	0.92 (0.33-2.57)
	90 to <183		7	0.52 (0.24-1.16)
	183 to <366		14	**0.53 (0.29-0.97)**
	Cerebral infarction	28	-	1
	-3 to <7		3	3.27 (0.99-10.8)
	7 to <14		1	1.56 (0.21-11.4)
	14 to <30		1	0.68 (0.09-5.00)
	30 to <60		6	2.18 (0.90-5.26)
	60- to <90		2	0.73 (0.17-3.05)
	90 to <183		4	0.47 (0.16-1.34)
	183 to <366		11	0.65 (0.33-1.32)
	**Intracerebral haemorrhage**	6	-	1
	-3 to <7		2	**10.2 (2.05-50.4)**
	7 to <14		0	nc
	14 to <30		0	nc
	30 to <60		1	1.69 (0.20-14.1)
	60- to <90		1	1.69 (0.20-14.1)

	Condition	Baseline	Post-exposure	IRR (95% CI)
	90 to <183		2	1.09 (0.22-5.42)
	183 to <366		2	0.56 (0.11-2.75)
	Transitory cerebral ischaemic attack	12	-	1
	-3 to <7		0	nc
	7 to <14		0	nc
	14 to <30		1	1.59 (0.21-12.2)
	30 to <60		1	0.85 (0.11-6.52)
	60- to <90		1	0.85 (0.11-6.52)
	90 to <183		2	0.55 (0.12-2.44)
	183 to <366		2	0.28 (0.06-1.24)
**Venous thromboembolic events**	**Venous thromboembolism (summary)**	14	-	1
	-3 to <7		1	2.18 (0.29-16.6)
	7 to <14		0	nc
	14 to <30		4	**5.45 (1.79-16.6)**
	30 to <60		6	**4.36 (1.67-11.3)**
	60- to <90		6	**4.36 (1.67-11.3)**
	90 to <183		6	1.41 (0.59-3.66)
	183 to <366		11	1.31 (0.59-2.88)
	Pulmonary embolism	10	-	1
	-3 to <7		1	3.05 (0.39-23.8)
	7 to <14		0	nc
	14 to <30		3	**5.72 (1.57-20.8)**
	30 to <60		5	**5.08 (1.74-14.9)**
	60- to <90		2	2.03 (0.45-9.28)
	90 to <183		4	1.31 (0.41-4.18)
	183 to <366		6	1.00 (0.36-2.75)
	Lower limb embolism, phlebitis and thrombophlebitis	4	-	1
	-3 to <7		0	nc
	7 to <14		0	nc
	14 to <30		1	4.77 (0.53-42.6)
	30 to <60		1	2.54 (0.28-22.7)
	60- to <90		3	**7.63 (1.71-34.1)**
	90 to <183		2	1.64 (0.30-8.95)
	183 to <366		6	2.50 (0.71-8.86)
	**Thrombocytopenia and coagulative disorders**	3	-	1
	-3 to <7		0	nc
	7 to <14		0	nc
	14 to <30		0	nc
	30 to <60		1	3.39 (0.35-32.6)
	60- to <90		0	nc
	90 to <183		0	nc
	183 to <366		2	1.11 (0.19-6.65)
	**Encephalitis, myelitis and encephalomyelitis**	3	-	1
	-3 to <7		0	nc
	7 to <14		0	nc

(Header: Period spans Baseline and Post-exposure)

		Period		
	Condition	Baseline	Post-exposure	IRR (95% CI)
	14 to <30		1	6.35 (0.66-61.1)
	30 to <60		0	nc
	60- to <90		0	nc
	90 to <183		0	nc
	183 to <366		0	nc
	**Bleeding events and anaemias**	64	-	1
	-3 to <7		4	1.91 (0.69-5.23)
	7 to <14		2	1.36 (0.33-5.56)
	14 to <30		5	1.49 (0.60-3.70)
	30 to <60		6	0.95 (0.41-2.20)
	60- to <90		6	0.95 (0.41-2.20)
	90 to <183		21	1.08 (0.66-1.76)
	183 to <366		24	0.65 (0.41-1.03)
	**Acute kidney failure**	19	-	1
	-3 to <7		1	1.61 (0.21-12.0)
	7 to <14		0	nc
	14 to <30		2	2.01 (0.47-8.61)
	30 to <60		3	1.61 (0.48-5.42)
	60- to <90		0	nc
	90 to <183		0	nc
	183 to <366		1	**0.09 (0.01-0.66)**
**Respiratory events**	**Respiratory events (summary)**	212	-	1
	-3 to <7		621	**89.3 (76.4-104)**
	7 to <14		326	**67.0 (56.4-79.7)**
	14 to <30		64	**5.75 (4.35-7.61)**
	30 to <60		24	1.15 (0.75-1.76)
	60- to <90		12	0.58 (0.32-1.03)
	90 to <183		44	**0.68 (0.49-0.94)**
	183 to <366		68	**0.53 (0.41-0.70)**
	Respiratory (infectious) events*	128	-	1
	-3 to <7		548	**131 (108-158)**
	7 to <14		284	**96.7 (78.5-119)**
	14 to <30		58	**8.64 (6.33-11.8)**
	30 to <60		12	0.95 (0.53-1.72)
	60- to <90		9	0.71 (0.36-1.41)
	90 to <183		14	**0.36 (0.21-0.62)**
	183 to <366		31	**0.40 (0.27-0.60)**
	Respiratory (non-infectious) events	52	-	1
	-3 to <7		58	**34.0 (23.4-49.5)**
	7 to <14		40	**33.5 (22.2-50.6)**
	14 to <30		11	**4.03 (2.10-7.73)**
	30 to <60		8	1.56 (0.74-3.29)
	60- to <90		7	1.37 (0.62-3.01)
	90 to <183		28	**1.77 (1.12-2.80)**
	183 to <366		36	1.15 (0.75-1.76)

	Condition	Period		
		Baseline	Post-exposure	IRR (95% CI)
	Respiratory (asthma) events	51	-	1
	-3 to <7		18	**10.8 (6.29-18.4)**
	7 to <14		10	**8.54 (4.34-16.8)**
	14 to <30		1	0.37 (0.05-2.70)
	30 to <60		5	1.00 (0.40-2.50)
	60- to <90		2	0.40 (0.10-1.64)
	90 to <183		9	0.58 (0.28-1.18)
	183 to <366		17	**0.56 (0.32-0.96)**
**Negative controls**	**Injury**	120	-	1
	-3 to <7		9	**2.29 (1.16-4.50)**
	7 to <14		1	0.36 (0.05-2.60)
	14 to <30		3	0.48 (0.15-1.50)
	30 to <60		8	0.68 (0.33-1.39)
	60- to <90		9	0.76 (0.39-1.50)
	90 to <183		26	0.71 (0.47-1.09)
	183 to <366		39	**0.54 (0.38-0.78)**
	**Urinary tract infection**	52	-	1
	-3 to <7		2	1.17 (0.29-4.82)
	7 to <14		1	0.84 (0.12-6.06)
	14 to <30		3	1.10 (0.34-3.52)
	30 to <60		3	0.59 (0.18-1.88)
	60- to <90		4	0.78 (0.28-2.16)
	90 to <183		6	**0.38 (0.16-0.88)**
	183 to <366		16	**0.51 (0.29-0.90)**

SARS-CoV-2 – Severe acute respiratory Syndrome Coronavirus 2; nc – not calculable; Conditions with a 'summary' category do not represent as sum of each component part, as individuals could have more than one coded condition of interest.

References

1. Government of Victoria. Public Health and Wellbeing Act (Act Number 46/2008). Authorised version incorporating amendments as at 30 Aug 2022. https://www.legislation.vic.gov.au/in-force/acts/public-health-and-wellbeing-act-2008/056 (viewed Sept 2022).

2. Victorian Department of Health. Department of Health information asset register extract. Updated Apr 2022. https://www.health.vic.gov.au/sites/default/files/2022-04/information-asset-register-extract.xlsx (viewed Aug 2022).

3. Victorian Department of Health. Victorian Admitted Episodes Dataset (VAED) manual 2020-2021. Updated 15 July 2022. https://www.health.vic.gov.au/data-reporting/victorian-admitted-episodes-dataset (viewed Sept 2022).

4. Government of Victoria. Birth Deaths and Marriages Registration Act (Act Number 43/1996). Authorised version incorporating amendments as at 1 May 2020. https://www.legislation.vic.gov.au/in-force/acts/births-deaths-and-marriages-registration-act-1996/039 (viewed Sept 2022).

5. Victorian Department of Health. The Centre for Victorian Data Linkage. Updated 3 Mar 2022. https://www.health.vic.gov.au/reporting-planning-data/the-centre-for-victorian-data-linkage (viewed Sept 2022).

6. Curtis SJ, Cutcher Z, Brett JA, et al. An evaluation of enhanced surveillance of hospitalised COVID-19 patients to inform the public health response in Victoria. *Commun Dis Intell (2018)* 2020; 44.

7. Independent Health and Aged Care Pricing Authority. How to classify COVID-19. Updated 1 July 2022. https://www.ihacpa.gov.au/health-care/classification/how-classify-covid-19 (viewed Sept 2022).

8. Safer Care Victoria. Victorian additions to Australian Coding Standards. Updated 1 Dec 2020. https://www.safercare.vic.gov.au/data-reports/clinical-coding-and-classifications/victorian-additions-to-australian-coding-standards (viewed Sept 2022).
